# MicroRNA signatures predict early major coronary events in middle-aged men and women

**DOI:** 10.1038/s41419-020-2291-9

**Published:** 2020-01-30

**Authors:** Bruna Gigante, Laura Papa, Anja Bye, Paolo Kunderfranco, Chiara Viviani, Roberta Roncarati, Carlo Briguori, Ulf de Faire, Matteo Bottai, Gianluigi Condorelli

**Affiliations:** 1 Cardiovascular Medicine Unit, Department of Medicine, Karolinska Institutet, Stockholm, Sweden; 2 Department of Cardiovascular Medicine, Humanitas Clinical and Research Center – IRCCS, Rozzano Milan, Italy; 3 Department of Cardiology, St. Olavs Hospital, Faculty of Medicine and Health Sciences, Norwegian University of Science and Technology, Trondheim, Norway; 4 Institute of Genetics and Biomedical Research, National Research Council of Italy, Rozzano Milan, Italy; 5 Interventional Cardiology Unit, Mediterranea Cardiocentro, Naples, Italy; 6 Unit of Cardiovascular and Nutritional Epidemiology, Institute of Environmental Medicine (IMM), Karolinska Institutet and Tema Coronary and Valvular Disease and Karolinska University Hospital, Stockholm, Sweden; 7 Unit of Biostatistics, IMM, Karolinska Institutet, Stockholm, Sweden; 8 Humanitas University, Pieve Emanuele, Milan Italy

**Keywords:** Predictive markers, miRNAs

Dear Editor,

MicroRNAs share many of the essential features of a good circulating biomarker^1^, but despite promising data on their role in risk prediction for major adverse coronary events (MACEs)^2,3^, more investigation is needed for translation to the clinic. Thus, we have sought to identify circulating microRNA signatures able to predict MACEs, defined as myocardial infarction (MI), angina, or sudden cardiac death (Supplementary Fig. I).

To this end, we obtained plasma from the first 100 MACE-presenting individuals who had been enrolled in the 60 year olds from Stockholm study (60YO)^4^, as well as from 100 MACE-free referents during an 11-year follow-up period (Supplementary Table I), and used the samples for a PCR-based method to screen 754 microRNAs (Supplementary Material). Of the 55 microRNAs with the greatest difference in expression in cases vs. referents (Supplementary Fig. II), microRNA-145-3p was found associated with the largest estimated risk increase (odds ratio [OR]: 2.18; 95% confidence interval [CI]: 1.27–3.75), while microRNA-720 was associated with reduced MACE risk (OR: 0.47; 95% CI: 0.24–0.92), after adjustment for common cardiovascular risk factors (Supplementary Table II). No correlation was observed for any of the 55 microRNAs with C-reactive protein or lipid levels (triglycerides, low-density lipoprotein-cholesterol, high-density lipoprotein-cholesterol). Then, because microRNAs can be pleiotropic and redundant, we performed an interaction analysis, identifying 16 microRNA pairs—constituting 16 microRNA signatures—in which microRNA-320b happened to be always present. The MACE risk associated with the interacting microRNA in the pair co-varied with the level of microRNA-320b: indeed, the risk associated with each microRNA was greatest at the highest microRNA-320b expression level (Fig. 1a).

**Fig. 1:**
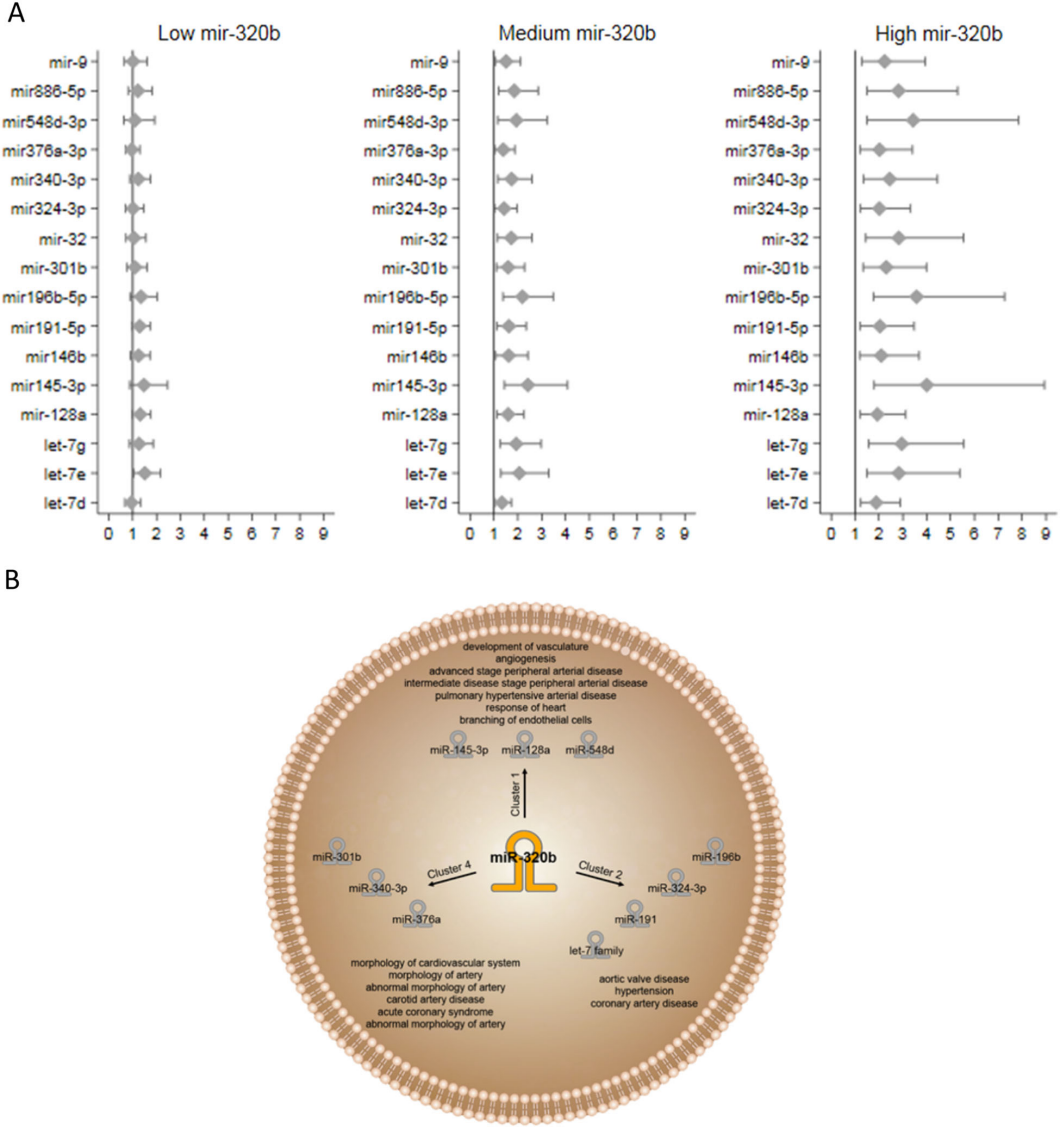
Association of 16 microRNAs interacting with microRNA-320b and the risk of MACE in the 60YO cohort, and functional interactions of microRNA-320b. **a** Increasing plasma expression of microRNA-320b modulates the risk of MACE for 16 other microRNAs. The risk of MACE is expressed as OR (diamond) with 95% CI (horizontal line). Expression of microRNA-320b was classified as low (≤25th percentile), medium (>25th–≤75th percentile), or high (>75th percentile). mir, microRNA. **b** Schematic representation of three clusters identified by unsupervised cluster analysis, and the predicted function of the genes regulated by the mircroRNAs in clusters 1, 2, and 4.

MicroRNA-320b and its 16 interacting microRNAs had a total of 492 putative targets: 248 were predicted for microRNA-320b as well as at least one other interacting microRNA, and were groupable into four clusters (Supplementary Fig. III). Gene ontology revealed that three clusters (1, 2, and 4) were statistically linked with cardiovascular system development and function, as well as with the regulation of inflammation, thrombosis, and lipid metabolism (Fig. 1b; Supplementary Table III).

We then validated findings on a cohort from the Nord-Trøndelag Health study^5^ (Supplementary Material). Analysis of single microRNAs revealed a pattern of association with the risk of MI similar to that associated with MACE in the 60YO cohort, with the exception of microRNA-320b, microRNA-324-3p, and microRNA-32-5p (Supplementary Table IV). Coherently with discovery findings, increasing expression levels of microRNA-320b associated with progressive increase of the MI risk estimates for microRNAs from clusters 2 and 4. In particular, the trend was similar to that observed in the 60YO cohort for five microRNAs of cluster 2 (microRNA-191-5p, microRNA-324-3p, microRNA-196b-5p, let-7d, and let-7g) and for two microRNAs of cluster 4 (microRNA-301b and microRNA-340) (Supplementary Table V).

Thus, we have identified microRNA signatures predicting the risk of early MACE in middle-aged men and women free from cardiovascular diseases (Supplementary Discussion). Of note, interaction analysis revealed a complex functional network that was not evident when the microRNAs were analyzed independently, with microRNA-320b—found downregulated by others in platelets of patients with MI^6^ or carotid atherosclerotic plaques^7^—acting as a major modulator of MACE risk.

## Supplementary information

Supplemental file
