# Supplementary material for: MicroRNA signatures predict early major coronary events in middle-aged men and women
Source: Cell Death Dis. 2020 Jan 30;11(1):74. doi: 10.1038/s41419-020-2291-9 (PMC6992779; doi:10.1038/s41419-020-2291-9)
Supplement: Supplementary file 1 — Supplemental file [file 41419_2020_2291_MOESM1_ESM.docx]

# **ONLINE SUPPLEMENTAL MATERIAL**

#

# **MicroRNA signatures predict early major coronary events in middle-aged men and women**

Bruna Gigante, M.D., Ph.D.,^1^ Laura Papa, Ph.D.,^2^ Anja Bye, Ph.D,^3^ Paolo Kunderfranco, Ph.D.,^2^ Chiara Viviani, Ph.D., ^2^ Roberta Roncarati, Ph.D.,^4^ Carlo Briguori, M.D., Ph.D,^5^ Ulf de Faire, M.D., Ph.D,^6^ Matteo Bottai, Ph.D,^7^ and Gianluigi Condorelli, M.D., Ph.D.^2,4,8^

^1^ Cardiovascular Medicine Unit, Department of Medicine, Karolinska Institutet, Stockholm, Sweden, ^2^Department of Cardiovascular Medicine, Humanitas Clinical and Research Center – IRCCS, Rozzano, Milan, Italy; ^3^ Department of Cardiology, St. Olavs Hospital, Faculty of Medicine and Health Sciences, Norwegian University of Science and Technology, Trondheim, Norway; ^4^Institute of Genetics and Biomedical Research, National Research Council of Italy, Rozzano, Milan, Italy;  ^5^ Interventional Cardiology Unit, Mediterranea Cardiocentro, Naples, Italy;  ^6^Unit of Cardiovascular and Nutritional Epidemiology, Institute of Environmental Medicine (IMM), Karolinska Institutet and Tema Coronary and Valvular Disease and Karolinska University Hospital, Stockholm, Sweden; ^7^ Unit of Biostatistics, IMM, Karolinska Institutet, Stockholm, Sweden; ^8^ Humanitas University, Pieve Emanuele, Milan, Italy.

# **SUPPLEMENTAL MATERIALS AND METHODS**

## Discovery cohort: the cohort of 60-year-old men and women from Stockholm (60YO)

Every third man and woman reaching 60 years of age between July 1^st^ 1997 and June 30^th^ 1998 and living in Stockholm County was selected from the Swedish population register and invited to participate in a health screening[^1^](#_ENREF_1)^,^[^2^](#_ENREF_2). A total of 4,232 subjects (2,039 men and 2,193 women) agreed to participate in the study (78% response rate). Anamnestic records were obtained through a self-administered questionnaire. A biobank was established for the collection and storage (at −80ºC) of whole blood, serum, and plasma samples. The cohort is linked annually to the cause-of-death register and the hospital discharge register in Sweden (100% follow-up) via the participants’ personal identification number, using codes of the International Classification of Diseases, 10^th^ revision (ICD-10).

The study was approved by the Regional Ethical Review Board at Karolinska Institutet, Stockholm, Sweden (reference number 96/308). All study participants gave their informed consent to be enrolled in the study.

For the present study, MACE was defined as sudden cardiac death (ICD I46), first-time myocardial infarction (MI) (ICD I21) or angina requiring hospitalization (ICD I20 and I25). We selected two groups of study participants: early MACE (n=100), defined as study participants linked to the first consecutive 100 MACE diagnoses recorded in the registries during the follow-up. Referents (n=100) were matched by sex and time of inclusion in the cohort. Referents were still MACE-free after 11 years of follow-up, according to the data available when the experimental part of this study was planned.

## Validation cohort: the Nord-Trøndelag Health Study (HUNT)

Main results were validated in individuals from the HUNT Study, fully described elsewhere[^3^](#_ENREF_3). To validate the microRNA signatures identified in the 60YO cohort, we selected study participants (n=118) aged 60±5 years in whom incident MI occurred within one year from initial blood sampling (n=58) and age- and sex-matched referents (n=60) who did not develop MI during the same period.

## RNA isolation and microRNA profiling of samples from the 60YO cohort

Total RNA was isolated from plasma samples (500 μL) collected in EDTA-treated tubes, using the microRNAeasy Mini Kit (Qiagen, Hilden, Germany), following the manufacturer’s instructions. To increase recovery of isolated RNA, 1μl of 1ng/μl tRNA was added in the phenol phase during extraction. To avoid limitation of RNA quantifications due to the very low concentration of microRNAs in plasma, all samples were eluted in 20 μl of RNase-free water. The absorbance of each eluate was then quantified on a Nanodrop 1000 spectrophotometer (Nanodrop, Wilmington (DE), USA), and the samples diluted to the minimum concentration of 4 ng/μl needed to perform the OpenArray Low Sample Input protocol with the TaqMan OpenArray microRNA Panel, which tests for 754 validated human microRNAs, as described in the Applied Biosystems website (Life Technologies Corporation, Carlsbad (CA), USA).

In brief, reverse transcription (RT) of mature microRNAs was carried out using Megaplex RT Primer Human Pools A and B version 3.0 (Life Technologies). The obtained cDNA was pre-amplified with Megaplex PreAmp Primer Human Pools (A and B) and further diluted 1:20 with 0.1X Tris-EDTA, pH 8.0. Diluted samples were mixed with TaqMan OpenArray Real-Time PCR Master Mix and loaded on the arrays, using the OpenArray AccuFill System (Life Technologies). The Freedom Evo 150 liquid handling system (Tecan Group Ltd. Männedorf, Switzerland) was used to reliably aliquot reaction mixtures and samples in all protocol steps. Negative controls were included in each reaction. RT thermocycler conditions followed the manufacturer’s standard protocol instructions, and the array output was automatically analysed by OpenArray Real Time qPCR Analysis Software supplied with the instrument. To check technical reproducibility, 15% of samples were run in duplicate. Raw data obtained as output from the OpenArray platform were normalized with endogenous references (U6, RNU48, and microRNA-16-5p), which were selected using NormFinder^[4](#_ENREF_4)^ and geNorm^[5](#_ENREF_5)^ algorithms.

## RNA isolation and microRNA profiling of samples from HUNT study individuals

For the validation of findings, total RNA was extracted from serum samples obtained from HUNT study individuals, as previously described[^6^](#_ENREF_6). RNA (2 μl) was reverse transcribed in 10 μl reactions using the microRNACURY LNA Universal RT microRNA PCR, Polyadenylation, and cDNA Synthesis Kit by Exiqon (Vedbaek, Denmark) on a set of 20 microRNAs (let-7d-5p, let-7e-5p, let-7g-5p, microRNA-128-1-5p, microRNA-145-3p, microRNA-146b-5p, microRNA-191-5p, microRNA-196b-5p, microRNA-301b, microRNA-320b, microRNA-324-3p, microRNA-32-5p, microRNA-340-3p, microRNA-376a-3p, microRNA-9-5p, microRNA-548d-3p, microRNA-362-5p, microRNA-454-3p, microRNA-744-3p, and microRNA-93-5p) identified as associated with MACE in the 60YO cohort.

In brief, cDNA was diluted 50x and assayed in 10 μl PCR reactions according to the manufacturer’s protocol; each microRNA was assayed once by qPCR on the microRNA Ready-to-Use PCR, Custom Pick and Mix Panel, using ExiLENT SYBR Green Master Mix. One duplicate for each RT reaction was run, and negative controls excluding template from the reverse transcription reaction were profiled like the samples. Amplification was performed in a LightCycler480 Real-Time PCR System (Roche, Basel, Switzerland) in 384-well plates. The amplification curves were analysed using Roche LC software. The reference microRNA microRNA-16-5p was used for expression normalization.

## Statistical analyses

Data are reported as median and interquartile range (IQR). Normalized microRNA expression in plasma was expressed in arbitrary units and used in all subsequent analyses. To estimate microRNA expression level, the ∆Ct method was used. We calculated the difference between cycle threshold (Ct) for each microRNA and the Ct for the microRNAs used for normalization.

We tested the equality of the distribution of the expression of each microRNA in cases *vs.* referents with the Wilcoxon’s rank-sum test. The Kolmogorov-Smirnov’s test was used to test if the p-values were uniformly distributed over the interval (0, 1).

The risk of MACE associated with the expression level of circulating microRNAs was measured by odds ratios (OR) and the associated 95% confidence interval (95% CI) estimated with random-effect logistic regression. In all the analyses, we used the normalized microRNA expression as exposure. Since microRNA expression is inversely related to the Ct threshold value, the negative Ct value was used in the analyses to facilitate interpretation of the results. MicroRNA expression was centred at its median and rescaled to have an inter-quartile range (i.e., the difference between the 25^th^ and the 75^th^ percentile) equal to one. Crude risk estimates were adjusted for sex, diabetes (glucose serum levels ≥7 mmol/L and/or treatment with hypoglycaemic drugs or insulin), hypertension (blood pressure values ≥140/90 mmHg and/or treatment with anti-hypertensive medications), hyperlipidaemia (fasting total serum cholesterol >5.0 and/or treatment for hyperlipidaemia and/or self-reporting), smoking (current or past smoking habit), and obesity (body mass index >30kg/m^2^). In the HUNT study, risk estimates were adjusted by age and sex for the multivariate analysis.

To identify interacting microRNA pairs, we estimated random-effect logistic regression models for the probability of being a case, including the two considered microRNAs and their interaction but no other covariates. The random effect was associated with the matched-group identifier. We observed that microRNA-320b was present in all interacting microRNA pairs associated with the risk of MACE. We then tested if the expression level of microRNA-320b modified the association of the paired microRNA with the risk of MACE. To this end, microRNA-320b expression levels were categorized as low (≤25^th^ percentile), medium (>25^th^ –≤75^th^ percentile), or high (>75^th^ percentile). We then performed a sensitivity analysis by dropping all the microRNAs with less than 100 observations, but the interaction analysis results and risk estimates did not change.

Correlation between circulating microRNAs and C-reactive protein or serum lipid levels was estimated by the Spearman’s rank correlation coefficients.

The analyses were performed using Stata version 15.

## Cluster analysis and identification of targets of the microRNA signatures

The microRNA target filter analysis of the Ingenuity Pathway Analysis (IPA) suite was used to find predicted targets[^7^](#_ENREF_7) for the identified microRNA signatures. Unsupervised cluster analysis was performed with Cluster 3.0 and data visualized with Tree View. Four main clusters were identified based on the maximum number of microRNA targets in each cluster. Gene ontology (GO) analysis was also performed with the IPA suite, and functions related to cardiovascular categories. A p-value <0.05 was used as the statistical threshold.

SUPPLEMENTAL **TABLES**

Supplemental **Table I.** Baseline characteristics of the individuals selected from the 60YO cohort and included in the analysis

|  | **MACE**  (N=100) | **Referents** (N=100) |
| --- | --- | --- |
| Men/Women (N) | 72/28 | 72/28 |
| Time to event (years) | 2.3 (0.15–4.7) | - |
| Risk factors (N) |  |  |
| Diabetes mellitus | 16 | 3 |
| Smoking | 34 | 20 |
| Hypercholesterolaemia | 52 | 32 |
| Hypertriglyceridaemia | 24 | 12 |
| Obesity | 25 | 18 |
| Hypertension | 52 | 32 |
| Biochemical parameters |  |  |
| Total cholesterol (mmol/L)* | 6.1 (5.5–6.7) | 5.7 (5.0–6.5) |
| LDL (mmol/L)* | 4.2 (3.5–4.8) | 3.7 (3.1–4.5) |
| HDL (mmol/L)* | 1.4 (1.0–1.5) | 1.4 (1.1–1.6) |
| CRP (mg/L) | 2.7 (1.4–4.7) | 1.7 (0.9–3.1) |

MACE, represented by the 100 individuals with the earliest MACE diagnosis during follow-up; referents are 100 individuals free of MACE during the 11-year follow-up. Values are median (interquartile range). Abbreviations: LDL, low density lipoprotein; HDL, high density lipoprotein; CRP, C-reactive protein. Missing values: LDL cholesterol, n=7; * to convert values to mg/dL, multiply by 38.67.

# **Supplemental Table II**. Association of single microRNAs with the risk of MACE in the 60YO cohort

|  | N | Univariate  analysis | | Multivariate analysis |
| --- | --- | --- | --- | --- |
| microRNA-145-3p | 159 | 1.88 (1.22–2.90) | 2.18 (1.27–3.75) | |
| microRNA-362-3p | 167 | 1.51 (1.10–2.08) | 1.42 (1.00–2.02) | |
| microRNA-454 | 179 | 1.42 (1.04–1.92) | 1.44 (1.04–2.00) | |
| microRNA-20b | 177 | 1.71 (1.10–2.67) | 1.88 (1.12–3.17) | |
| microRNA-196b | 177 | 1.46 (1.02–2.07) | 1.69 (1.14–2.52) | |
| microRNA-185 | 196 | 1.38 (0.99–1.91) | 1.46 (1.02–2.11) | |
| microRNA-301b | 195 | 1.32 (0.96–1.81) | 1.58 (1.05–2.38) | |
| microRNA-19b1 | 71 | 2.05 (1.10–3.81) | 2.39 (1.11–5.17) | |
| microRNA-134 | 140 | 1.38 (0.97–1.94) | 1.54 (1.02–2.32) | |
| microRNA-720 | 154 | 0.55 (0.34–0.89) | 0.47 (0.24–0.92) | |
| microRNA-98 | 57 | 1.37 (0.94–1.98) | 1.74 (0.99–3.04) | |
| microRNA-542-3p | 108 | 2.07(1.14–3.77) | 2.18 (0.98–4.86) | |
| microRNA-9 | 191 | 1.41 (1.03–1.91) | 1.42 (0.98–2.05) | |
| let-7d | 183 | 1.23 (0.99–1.53) | 1.27 (0.98–1.64) | |
| microRNA-125a-5p | 125 | 1.52 (0.99 2.33) | 1.48 (0.97–2.25) | |
| microRNA-505-5p | 122 | 1.29 (0.91–1.82) | 1.43 (0.97–2.12) | |
| microRNA-338-3p | 166 | 1.49 (0.96–2.34) | 1.63 (0.97–2.73) | |
| microRNA-18a-3p | 186 | 1.47 (0.98–2.19) | 1.55 (0.96–2.50) | |
| microRNA-886-5p | 194 | 1.32 (0.93–1.86) | 1.45 (0.96–2.19) | |
| microRNA-93-5p | 194 | 1.46 (1.05–2.04) | 1.37 (0.95–1.98) | |
| microRNA-502-3p | 182 | 1.40 (0.99–1.99) | 1.31 (0.93–1.84) | |
| microRNA-31 | 111 | 1.22 (0.84–1.77) | 1.41 (0.93–2.12) | |
| microRNA-128a | 197 | 1.17 (0.91–1.49) | 1.22 (0.92–1.64) | |
| microRNA-744-3p | 161 | 1.46 (1.01–2.12) | 1.38 (0.91–2.09) | |
| microRNA-126 | 193 | 1.09 (0.91–1.31) | 1.11 (0.91–1.37) | |
| microRNA-548d-3p | 173 | 1.58 (1.01–2.49) | 1.57 (0.90–2.74) | |
| let-7g | 193 | 1.27 (0.91–1.75) | 1.29 (0.89–1.88) | |
| let-7e | 179 | 1.20 (0.87–1.65) | 1.26 (0.88–1.79) | |
| microRNA-324-3p | 180 | 1.19 (0.91–1.57) | 1.21 (0.88–1.65) | |
| microRNA-191-5p | 166 | 1.14 (0.88–1.47) | 1.17 (0.87–1.57) | |
| microRNA-106a | 184 | 1.04 (0.87-1.25) | 1.05 (0.86-1.29) | |
| microRNA-769-5p | 133 | 1.37 (0.94–2.01) | 1.36 (0.86–2.15) | |
| microRNA-222 | 145 | 1.54 (1.00–2.35) | 1.39 (0.85–2.27) | |
| microRNA-32-5p | 190 | 1.23 (0.90-1.67) | 1.19 (0.85-1.68) | |
| microRNA-423-5p | 195 | 1.19 (0.89–1.57) | 1.18 (0.85–1.65) | |
| microRNA-146b-5p | 197 | 1.12 (0.84–1.48) | 1.17 (0.83–1.65) | |
| microRNA-29b2 | 129 | 1.51 (0.99–2.30) | 1.35 (0.81–2.25) | |
| microRNA-376a-3p | 190 | 1.12 (0.89–1.42) | 1.04 (0.81–1.35) | |
| let-7b | 65 | 1.46 (0.87–2.43) | 1.44 (0.79–2.62) | |
| microRNA-106b-5p | 112 | 1.73 (1.01–2.97) | 1.42 (0.77–2.62) | |
| microRNA-340-3p | 192 | 1.21 (0.91–1.62) | 1.10 (0.77–1.57) | |
| microRNA-625 | 163 | 1.42 (0.95–2.13) | 1.22 (0.76–1.94) | |
| microRNA-362-3p | 147 | 1.37 (0.86–2.18) | 1.25 (0.73–2.13) | |
| microRNA-301 | 160 | 1.13 (0.82–1.57) | 1.04 (0.71–1.54) | |
| microRNA-122 | 171 | 1.39 (0.93-2.07) | 1.02 (0.63-1.65) | |
| microRNA-645 | 134 | 0.72 (0.49–1.06) | 0.98 (0.61–1.57) | |
| microRNA-206 | 181 | 0.80 (0.61-1.06) | 0.81 (0.58-1.12) | |
| microRNA-320b | 194 | 0.69 (0.49–0.96) | 0.75 (0.52–1.09) | |
| microRNA-184 | 111 | 0.74 (0.48–1.16) | 0.77 (0.46–1.28) | |
| microRNA-99-5p | 87 | 0.73 (0.47–1.13) | 0.75 (0.44–1.28) | |
| microRNA-664 | 122 | 0.45 (0.25–0.83) | 0.61 (0.30–1.23) | |
| microRNA-449b | 78 | 0.57 (0.29–1.09) | 0.66 (0.30–1.45) | |
| microRNA-1291 | 166 | 0.71 (0.47–1.05) | 0.61 (0.29–1.28) | |
| microRNA-127-4a | 148 | 0.66 (0.39–1.11) | 0.58 (0.28–1.18) | |
| microRNA-1249 | 79 | 0.49 (0.25–0.98) | 0.52 (0.23–1.16) | |

Associations with MACE risk estimated by logistic regression analysis and expressed as OR with 95% CI. Univariate analysis: association of single microRNAs with the risk of MACE. Multivariate logistic regression analysis: association of single microRNAs with the risk of MACE after adjustment for common cardiovascular risk factors (sex, diabetes, hypertension, hyperlipidaemia, and smoking), as defined in the Methods.

Nine microRNAs listed at the top of the table (microRNA-145-3p, microRNA-362-3p, microRNA-454, microRNA-20b, microRNA-196b, microRNA-185, microRNA-301b, microRNA-19b1 and microRNA-134) were associated with an increased risk of MACE, while one microRNA (microRNA-720) was associated with a reduced MACE risk.

MACE: major coronary event; N: number of individuals in which the microRNA was successfully amplified.

# **Supplemental Table III**. Gene Ontology Terms enriched in the four microRNA clusters.

|  | Functional Annotations | Predicted targets | *P*-value | |
| --- | --- | --- | --- | --- |
| **Cluster 1** | (51 predicted targets) |  |  | |
|  | Development of vasculature | *BMPR1A, CXCL2, EGR3, GLRX, HOXA5, KITLG, MTDH, SMAD9, VHL* | 2.96E-03 |  |
|  | Angiogenesis | *BMPR1A, CXCL2*, *EGR3*, *GLRX*, *HOXA5*, *KITLG*, *MTDH*, *VHL* | 4.11E-03 |  |
|  | Advanced stage PAD | *GLRX*, *MPPED2*, *PDLIM5* | 5.62E-03 |  |
|  | Pulmonary hypertension | *BMPR1A*, *SMAD9* | 6.23E-03 |  |
|  | Intermediate stage PAD | *GLRX*, *MPPED2*, *PDLIM5* | 6.51E-03 |  |
|  | Response of heart | *ASPH*, *BNIP3* | 8.15E-03 |  |
|  | Branching of endothelial cells | *KITLG*, *VHL* | 8.92E-03 |  |
| **Cluster 2** | (19 predicted targets) |  |  |  |
|  | Aortic valve disease | *GABRA5*, *PBX3* | 2.40E-03 |  |
|  | Hypertension | *GABRA5*, *PBX3*, *PRELID2* | 4.37E-02 |  |
|  | Coronary heart disease | *GABRA5*, *PBX3* | 4.46E-02 |  |
| **Cluster 3** | (31 predicted targets) |  |  |  |
|  | No CV annotations | - | - |  |
| **Cluster 4** | (27 predicted targets) |  |  |  |
|  | Morphology of artery | *RAC1*, *RASA1*, *SDHD* | 1.24E-03 |  |
|  | Carotid artery disease | *AKT3*, *SDHD* | 3.83E-03 |  |
|  | Abnormal Morphology of artery | *RASA1*, *SDHD* |  |  |
|  | Morphology of CV system | *AKT3*, *RAC1*, *RASA1*, *SDHD* | 3.67E-02 |  |
|  | Acute coronary syndromes | *GCG*, *RAC1* | 4.78E-02 |  |

Cluster 1: microRNA-320b plus microRNA-145-3p, microRNA-128a, microRNA-548d-3p; Cluster 2: microRNA-320b plus let-7g-5p, let-7d-5p, let-7e-5p, microRNA-196b-5p, microRNA-191-5p, microRNA-324-3p; Cluster 3: microRNA-320b plus microRNA-146b-5p, microRNA-9, microRNA-885-5p, microRNA-32; and Cluster 4: microRNA-320b plus microRNA-301b,microRNA-340-3p, microRNA-376a.

Abbreviations: PAD: peripheral artery disease, CV: cardiovascular.

Bone morphogenetic protein receptor 1A *(BMPR1A)*, Chemokine (C-X-C motif) ligand 2 (*CXCL2)*, Early Growth Response 3 *(EGR3)*, Glutaredoxin *(GLRX)*, Homeobox A5 *(HOXA5)*, KIT Ligand *(KITLG)*, Metadherin *(MTDH),* Mothers against decapentaplegic homolog 9 *(SMAD9),* von Hippel–Lindau *(VHL),* Metallophosphoesterase Domain Containing 2 *(MPPED2),* PDZ and LIM domain protein 5 *(PDLIM5),* Aspartate Beta-Hydroxylase *(ASPH),* BCL2 Interacting Protein 3 *(BNIP3),* Gamma-Aminobutyric Acid Type A Receptor Alpha5 Subunit *(GABRA5),* PBX Homeobox 3 *(PBX3),* PRELI domain containing 2 *(PRELID2),* Rac family small GTPase 1 *(RAC1),* RAS p21 protein activator 1 *(RASA1),* Succinate Dehydrogenase Complex Subunit D *(SDHD),* AKT serine/threonine kinase 3 *(AKT3),* Glucagon *(GCG).*

**Supplemental Table IV.** Association of 16 microRNAs – identified in the 60Y0 cohort as part of signatures associated with risk of MACE – with the risk of MI in the HUNT study.

|  | N | Univariate analysis | Multivariate analysis |
| --- | --- | --- | --- |
| microRNA-145-3p | 58 | 1.57 (0.22–11.17) | 1.51 (0.21–10.69) |
| microRNA-362 | 90 | 2.05 (0.29–14.54) | 2.10 (0.30–14.93) |
| let-7d | 117 | 1.47 (0.21–10.43) | 1.46 (0.21–10.38) |
| let-7e | 115 | 1.72 (0.24–12.23) | 1.74 (0.25–12.37) |
| let-7g | 117 | 1.79 (0.25–12.74) | 1.78 (0.25–12.65) |
| microRNA-146b-5p | 94 | 1.54 (0.22–10.94) | 1.48 (0.21–10.51) |
| microRNA-191-5p | 118 | 1.45 (0.20 –10.32) | 1.45 (0.20–10.30) |
| microRNA-196b-5p | 37 | 1.64 (0.23–11.67) | 2.60 (0.37–18.47) |
| microRNA-301b | 29 | 1.64 (0.23–11.64) | 1.90 (0.27–13.46) |
| microRNA-320b | 118 | 1.91 (0.27–13.55) | 1.91 (0.27–13.55) |
| microRNA-324-3p | 103 | 0.90 (0.13–6.42) | 0.90 (0.13–6.41) |
| microRNA-32-5p | 116 | 0.50 (0.07–3.58) | 0.50 (0.07–3.57) |
| microRNA-340-3p | 59 | 1.48 (0.2–10.52) | 1.48 (0.21–10.54) |
| microRNA-376a-3p | 108 | 1.10 (0.16–7.83) | 1.12 (0.16–7.96) |
| microRNA-454-3p | 110 | 1.68 (0.24–11.93) | 1.67 (0.24–11.89) |
| microRNA-93-5p | 117 | 1.65 (0.23–11.73) | 1.64 (0.23–11.65) |

Out of 20 microRNAs amplified in the HUNT study, four (i.e., microRNA-128a-5p, microRNA-9-5p, microRNA-548d-3p and microRNA-744-3p) were not successfully amplified, leaving the 16 microRNAs reported in the table above to be analysed for association with MI. Associations estimated by logistic regression analysis and expressed as OR with 95% CI.

**Supplemental Table V.** Myocardial infarction risk estimates for each interacting microRNA at different microRNA-320b expression ranges

|  | Low  microRNA-320b | Medium microRNA-320b | High  microRNA-320b | |
| --- | --- | --- | --- | --- |
| Cluster 1 |  |  | |  |
| microRNA-145-3p | 1.56 (0.49–4.93) | 1.16 (0.57**–**2.38) | | 0.87 (0.26**–**2.93) |
| Cluster 2 |  |  | |  |
| microRNA-191-5p | 0.65 (0.26–1.62) | 0.95 (0.48–1.89) | | 1.40 (0.48–4.10) |
| microRNA-196b-5p | 0.27 (0.03–2.63) | 1.47 (0.49–4.46 ) | | 8.21 (0.68–99.65) |
| microRNA-324-3p | 0.29 (0.08–1.08) | 0.77 (0.44–1.37 ) | | 2.06 (0.61–6.93) |
| let-7g | 0.47 (0.16–1.38) | 1.59 (0.86–2.92) | | 5.30 (1.52–18.47) |
| let-7d | 0.78 (0.30-2.02) | 1.09 (0.60-1.98) | | 1.53 (0.59-3.97) |
| let-7e | 2.15 (0.69-6.68) | 1.64 (0.94-2.85) | | 1.25 (0.53-2.96) |
| Cluster 3 |  |  | |  |
| microRNA-325p | 0.28 (0.10**–**0.76) | 0.44 (0.26**–**0.75) | | 0.71 (0.33**–**1.53) |
| microRNA-146b-5p | 1.56 (0.49**–**4.93) | 1.16 (0.57**–**2.38) | | 0.87 (0.26**–**2.93) |
| Cluster 4 |  |  | |  |
| microRNA-301b | 1.53(0.06**–**38.40) | 2.30 (0.62**–**8.60) | | 3.47 (0.31**–**38.36) |
| microRNA-340-3p | 0.85 (0.22–3.27) | 1.08 (0.46**–**2.51) | | 1.38 (0.29**–**6.59) |
| microRNA-376a-3p | 0.98 (0.34–2.79) | 0.86 (0.47**–**1.56) | | 0.76 (0.24**–**2.38) |

Association of microRNAs identified as interacting with microRNA-320b in the four clusters identified in the 60YO cohort. Risk of MI was estimated by logistic regression analysis and is expressed as OR (95% CI). microRNA-320b expression levels stratified into low (≤25th percentile), medium (>25th –≤75th percentile), and high (>75th percentile).

# **SUPPLEMENTAL FIGURES**

#
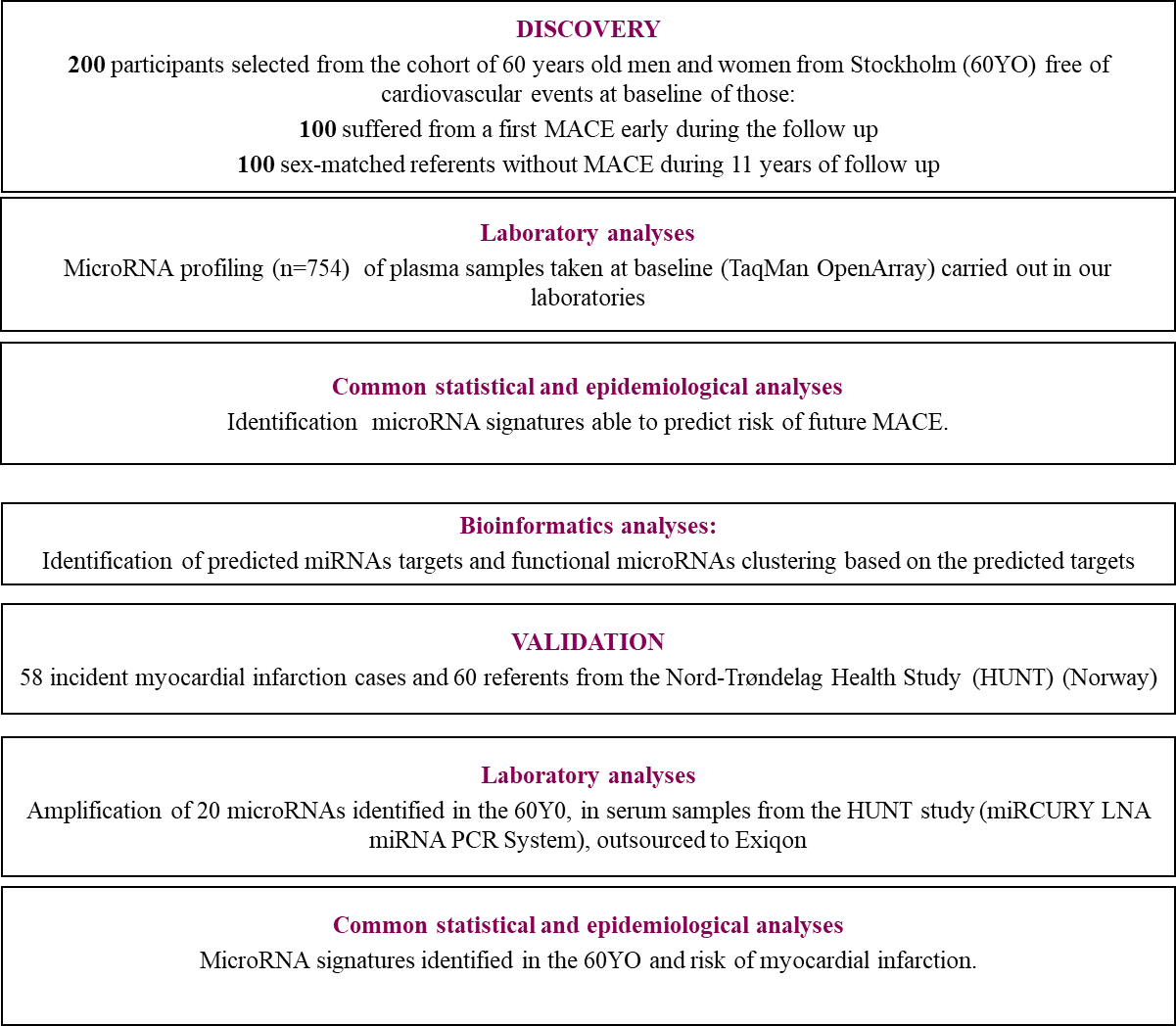


# **Supplemental Figure I.** Study design flowchart.


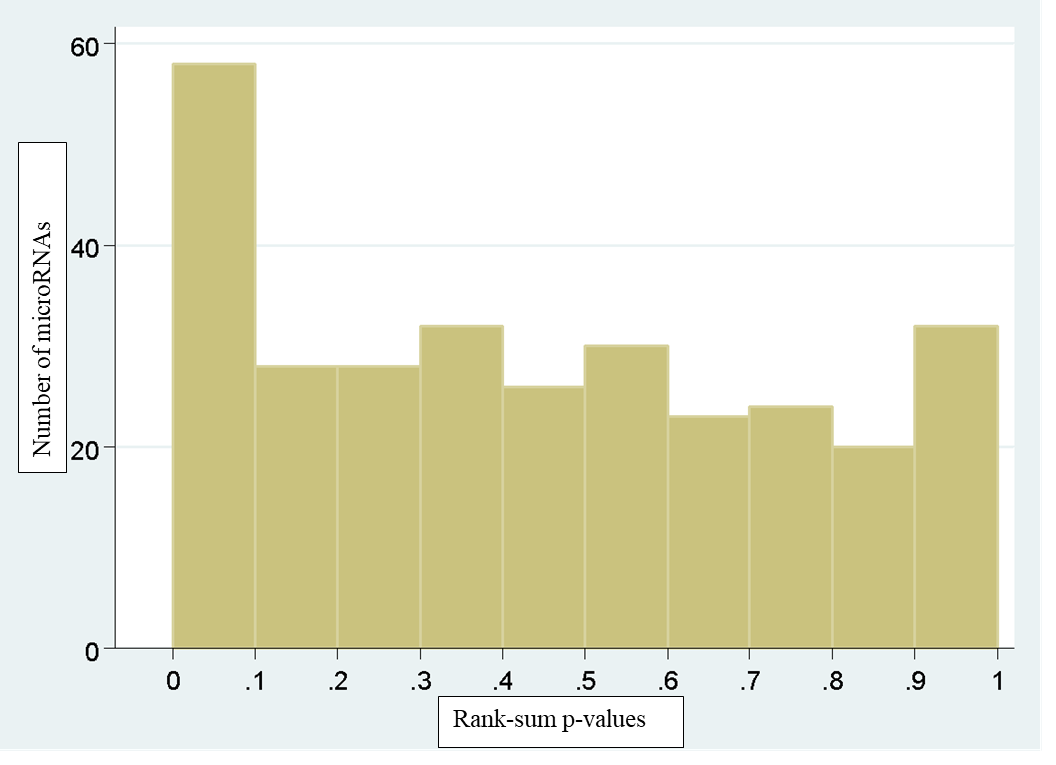


**Supplemental Figure II**. **Fifty-five microRNAs show the greatest difference in expression in cases vs. referents.** Out of the 754 microRNAs potentially assessable, only 301 (40%) could be amplified. The histogram summarizes the results of the Wilcoxon´s rank-sum test. The test was used to identify microRNAs showing the difference in expression in cases vs. referents, with below the arbitrary cut-off of rank-sum p value 0.1. Fifty-eight microRNAs out of 301 (first column in the histogram) were ranked with a p-value < 0.1; three were successfully amplified in less than 50 individuals and were excluded; 55 microRNAs were further analysed for association with risk of MACE.


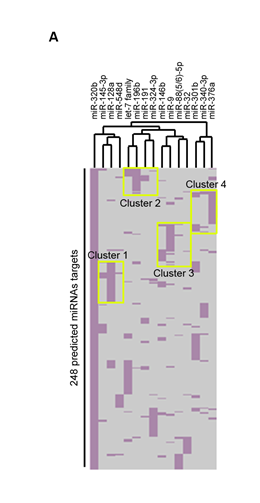


**Supplemental Figure III**. **Heatmap of hierarchical clustering of microRNA targets.** Overall, 492 predicted microRNA targets were identified, but only those targeted by microRNA-320b as well as by at least one other microRNA are shown (n=248). Highlighted in yellow are four main clusters formed from the interaction of microRNA-320b with the following microRNAs: microRNA-145-3p, microRNA-128a, microRNA-548d-3p, let-7 family, microRNA-196b-5p, microRNA-191-5p, microRNA-324-3p, microRNA-146b-5p, microRNA-9, microRNA-88(5/6)**-**5p, microRNA-32, microRNA-301b, microRNA-340-3p, and microRNA-376a-3p.

# **SUPPLEMENTAL DISCUSSION**

The analysis of the interaction between microRNAs revealed a complex functional network that was not evident when microRNAs were independently analysed for association with MACE. In particular, we identified microRNA-320b as a main modulator of the MACE risk: a high microRNA-320b expression level almost doubled the risk of MACE associated with the interacting microRNAs.

Biogenesis and maturation of microRNA-320b follow the non-canonical pathway [^9^](#_ENREF_9). It is known to be released from platelets upon thrombin stimulation and to downregulate intercellular adhesion molecule expression in endothelial cells, exerting an anti-inflammatory effect[^10^](#_ENREF_10). Observational studies have shown that microRNA-320b expression is downregulated in platelets of patients with MI[^10^](#_ENREF_10) or carotid atherosclerotic plaques[^11^](#_ENREF_11). These findings are in line with our univariate analysis of the 60YO cohort, in whom increased plasma expression of microRNA‑320b is associated with a reduced risk of MACE. In contrast, lower microRNA-320b was positively associated with MI in a Chinese population[^12^](#_ENREF_12), as reported here for the HUNT study cohort. These contrasting results highlight the difficulty in reproducing association analyses of single microRNAs[^13^](#_ENREF_13).

Several of the microRNAs reported here to interact with microRNA-320b have been previously described as associated with the atherosclerotic process[^14-17^](#_ENREF_14). For instance, microRNA‑145-3p – statistically assigned to cluster 1 – is known to regulate the switching of smooth muscle cells from a contractile to a proliferative phenotype[^11^](#_ENREF_11), the cycling of smooth muscle cells through UHRF1 (ubiquitin-like, containing PHD and RING finger domains, 1), an enzyme involved in DNA methylation[^18^](#_ENREF_18), and to affect endothelial function through cell–cell communication[^19^](#_ENREF_19)^,^[^20^](#_ENREF_20).

MicroRNAs statistically assigned to cluster 2 and interacting with microRNA-320b have been previously studied in *in vitro* and *ex vivo* studies and have been reported to regulate inflammation and thrombosis, two key mechanisms for atherosclerosis progression. In particular, microRNA-191-5p was found to exert a pro-inflammatory effect through the activation of NF-kb in endothelial cells[^21^](#_ENREF_21), to be highly expressed in microparticles derived from endothelial cells in smokers[^22^](#_ENREF_22), and its expression in platelets to be downregulated by treatment with aspirin[^23^](#_ENREF_23). In the 60YO cohort, microRNA-191-5p was highly correlated with microRNA-126 (r2>0.8), which is reported to modulate pathways involved in diabetic cardiomyopathy[^24^](#_ENREF_24). Moreover, microRNA‑324-3p – which was highly correlated with let-7d in the discovery cohort – and microRNA-196b are reportedly involved in the regulation of inflammatory processes in the intestinal mucosa[^25^](#_ENREF_25). Finally, let-7g – a highly conserved microRNA expressed in endothelial cells and smooth muscle cells – has been shown to modulate the atherosclerotic process in an experimental animal model of the disease[^21^](#_ENREF_21). In the HUNT study, let-7g was found associated with risk of fatal MI[^6^](#_ENREF_6). Cluster 2 microRNAs were found to regulate the expression of three target genes, one of which (namely, *PRELID2*) has been associated with the risk of coronary heart disease[^27^](#_ENREF_27). Finally, cluster 4 microRNAs have been reported involved in the regulation of interleukin 1β, an upstream regulator of the central axis of inflammation in atherosclerosis[^28^](#_ENREF_28), and microRNA-301b has been shown to regulate lipid metabolism[^29^](#_ENREF_29).

The same trend of association with changes in risk estimates according to microRNA‑320b expression levels was observed in the HUNT study for five out of the six interacting microRNAs belonging to cluster 2 (microRNA-191-5p, microRNA-324-3p, let-7g-5p, let-7d-5p, and microRNA 196b-5p) and for two out of the three microRNAs interacting in cluster 4 (microRNA-301b and microRNA-340-3p).

Several methodological differences in the study designs used for the two cohorts – including the use of different biological specimens (plasma *vs*. serum), different analytical methods (circulating microRNA levels were assessed at our laboratories for the 60YO cohort but outsourced to a biotech company for the HUNT individuals) and normalization strategies, two slightly different outcomes (MI, angina, and sudden cardiac death *vs*. MI alone), and follow-up times – may well contribute to explain the broader confidence interval and the lack of formal statistical significance in the HUNT study.

Our study is hypothesis generating and has strengths and limitations. It was designed to overcome differences in the expression of microRNAs with age, since all individuals were 60 years old at baseline (age was also a criterion used to select individuals for the validation phase). On the other hand, the generalizability of our results could be limited by this age specificity as well as by the specific genetic background of the participants and the environmental factors they were subjected to (all were living in Scandinavia), factors that probably affect microRNA expression. Given the observational nature of our study, we could not describe the mechanisms underlying the observed effect of microRNA-320b on the risk for future MACE; mechanistic studies are warranted to better define the role of this microRNA. In the bioinformatics approach used in the present study, the same seed recognized all microRNAs belonging to let-7 family members, including also microRNAs not analysed in the present study: thus, we could not better define the role of the single components belonging to this cluster 2 microRNA family. In addition, we could not successfully amplify all the microRNAs assigned to cluster 1 in the HUNT study participants and, therefore, we could not fully replicate the findings obtained with the 60YO cohort. Finally, the use of an array containing only 754 microRNAs might have precluded discovery of other microRNAs potentially associated with the risk of MACE, although measurable microRNAs in plasma are in the order of two to three hundreds.

In conclusion, we have identified novel microRNA signatures comprising microRNAs

involved in the regulation of lipid metabolism, inflammation, and thrombosis, that associate with and predict the risk of MACE. This finding highlights the potential of microRNAs as novel predictors of future MACE in aged populations.

# **SUPPLEMENTAL REFERENCES**

1. Ziegler L, Gajulapuri A, Frumento P, Bonomi A, Wallen H, de Faire U*, et al.* Interleukin 6 trans-signalling and risk of future cardiovascular events. *Cardiovasc Res* 2019; **115:** 213-221.

2. Gigante B, Leander K, Vikstrom M, Frumento P, Carlsson AC, Bottai M*, et al.* Elevated ApoB serum levels strongly predict early cardiovascular events. *Heart* 2012; **98:** 1242-1245.

3. Krokstad S, Langhammer A, Hveem K, Holmen TL, Midthjell K, Stene TR*, et al.* Cohort Profile: the HUNT Study, Norway. *Int J Epidemiol* 2013; **42:** 968-977.

4. Andersen CL, Jensen JL, Ørntoft TF. Normalization of real-time quantitative reverse transcription-PCR data: a model-based variance estimation approach to identify genes suited for normalization, applied to bladder and colon cancer data sets. *Cancer Res* 2004; **64:** 5245-5250.

5. Vandesompele J, De Preter K, Pattyn F, Poppe B, Van Roy N, De Paepe A*, et al.* Accurate normalization of real-time quantitative RT-PCR data by geometric averaging of multiple internal control genes. *Genome Biol* 2002; **3:** RESEARCH0034.

6. Bye A, Rosjo H, Nauman J, Silva GJ, Follestad T, Omland T*, et al.* Circulating microRNAs predict future fatal myocardial infarction in healthy individuals - The HUNT study. *J Mol Cell Cardiol* 2016; **97:** 162-168.

7. Kramer A, Green J, Pollard J, Jr., Tugendreich S. Causal analysis approaches in Ingenuity Pathway Analysis. *Bioinformatics* 2014; **30:** 523-530.

8. D'Agostino RB, Sr., Vasan RS, Pencina MJ, Wolf PA, Cobain M, Massaro JM*, et al.* General cardiovascular risk profile for use in primary care: the Framingham Heart Study. *Circulation* 2008; **117:** 743-753.

9. Condorelli G, Latronico MV, Cavarretta E. microRNAs in cardiovascular diseases: current knowledge and the road ahead. *J Am Coll Cardiol* 2014; **63:** 2177-2187.

10. Gidlof O, van der Brug M, Ohman J, Gilje P, Olde B, Wahlestedt C*, et al.* Platelets activated during myocardial infarction release functional miRNA, which can be taken up by endothelial cells and regulate ICAM1 expression. *Blood* 2013; **121:** 3908-3917, s3901-3926.

11. Zhang R, Qin Y, Zhu G, Li Y, Xue J. Low serum miR-320b expression as a novel indicator of carotid atherosclerosis. *J Clin Neurosci* 2016; **33:** 252-258.

12. Huang S, Chen M, Li L, He M, Hu D, Zhang X*, et al.* Circulating MicroRNAs and the occurrence of acute myocardial infarction in Chinese populations. *Circ Cardiovasc Genet* 2014; **7:** 189-198.

13. Schwarzenbach H, da Silva AM, Calin G, Pantel K. Data Normalization Strategies for MicroRNA Quantification. *Clin Chem* 2015; **61:** 1333-1342.

14. Barwari T, Rienks M, Mayr M. MicroRNA-21 and the Vulnerability of Atherosclerotic Plaques. *Mol Ther* 2018; **26:** 938-940.

15. Boon RA, Dimmeler S. MicroRNA-126 in atherosclerosis. *Arterioscler Thromb Vasc Biol* 2014; **34:** e15-16.

16. Cipollone F, Felicioni L, Sarzani R, Ucchino S, Spigonardo F, Mandolini C*, et al.* A unique microRNA signature associated with plaque instability in humans. *Stroke* 2011; **42:** 2556-2563.

17. De Rosa R, De Rosa S, Leistner D, Boeckel JN, Keller T, Fichtlscherer S*, et al.* Transcoronary Concentration Gradient of microRNA-133a and Outcome in Patients With Coronary Artery Disease. *Am J Cardiol* 2017; **120:** 15-24.

18. Elia L, Kunderfranco P, Carullo P, Vacchiano M, Farina FM, Hall IF*, et al.* UHRF1 epigenetically orchestrates smooth muscle cell plasticity in arterial disease. *J Clin Invest* 2018; **128:** 2473-2486.

19. Hergenreider E, Heydt S, Treguer K, Boettger T, Horrevoets AJ, Zeiher AM*, et al.* Atheroprotective communication between endothelial cells and smooth muscle cells through miRNAs. *Nat Cell Biol* 2012; **14:** 249-256.

20. Climent M, Quintavalle M, Miragoli M, Chen J, Condorelli G, Elia L. TGFbeta Triggers miR-143/145 Transfer From Smooth Muscle Cells to Endothelial Cells, Thereby Modulating Vessel Stabilization. *Circ Res* 2015; **116:** 1753-1764.

21. Liu M, Tao G, Liu Q, Liu K, Yang X. MicroRNA let-7g alleviates atherosclerosis via the targeting of LOX-1 in vitro and in vivo. *Int J Mol Med* 2017; **40:** 57-64.

22. Serban KA, Rezania S, Petrusca DN, Poirier C, Cao D, Justice MJ*, et al.* Structural and functional characterization of endothelial microparticles released by cigarette smoke. *Sci Rep* 2016; **6:** 31596.

23. Mayr M, Zampetaki A, Willeit P, Willeit J, Kiechl S. MicroRNAs within the continuum of postgenomics biomarker discovery. *Arterioscler Thromb Vasc Biol* 2013; **33:** 206-214.

24. Rawal S, Munasinghe PE, Shindikar A, Paulin J, Cameron V, Manning P*, et al.* Down-regulation of proangiogenic microRNA-126 and microRNA-132 are early modulators of diabetic cardiac microangiopathy. *Cardiovasc Res* 2017; **113:** 90-101.

25. Fasseu M, Treton X, Guichard C, Pedruzzi E, Cazals-Hatem D, Richard C*, et al.* Identification of restricted subsets of mature microRNA abnormally expressed in inactive colonic mucosa of patients with inflammatory bowel disease. *PLoS One* 2010; **5**.

26. Jiao M, You HZ, Yang XY, Yuan H, Li YL, Liu WX*, et al.* Circulating microRNA signature for the diagnosis of childhood dilated cardiomyopathy. *Sci Rep* 2018; **8:** 724.

27. Talmud PJ, Drenos F, Shah S, Shah T, Palmen J, Verzilli C*, et al.* Gene-centric association signals for lipids and apolipoproteins identified via the HumanCVD BeadChip. *Am J Hum Genet* 2009; **85:** 628-642.

28. Ceneri N, Zhao L, Young BD, Healy A, Coskun S, Vasavada H*, et al.* Rac2 Modulates Atherosclerotic Calcification by Regulating Macrophage Interleukin-1beta Production. *Arterioscler Thromb Vasc Biol* 2017; **37:** 328-340.

29. Wagschal A, Najafi-Shoushtari SH, Wang L, Goedeke L, Sinha S, deLemos AS*, et al.* Genome-wide identification of microRNAs regulating cholesterol and triglyceride homeostasis. *Nat Med* 2015; **21:** 1290-1297.
